# Sociodemographic associations of geographic variation in paediatric tonsillectomy and adenoidectomy

**DOI:** 10.1038/s41598-021-95522-5

**Published:** 2021-08-05

**Authors:** Aimy H. L. Tran, Danny Liew, Rosemary S. C. Horne, Joanne Rimmer, Gillian M. Nixon

**Affiliations:** 1 Department of Paediatrics, The Ritchie Centre, Hudson Institute of Medical Research, Monash University, Melbourne, Australia; 2 School of Public Health and Preventive Medicine, Monash University, Melbourne, Australia; 3 Department of Otolaryngology, Head and Neck Surgery, Monash Health, Melbourne, Australia; 4 Department of Surgery, Monash University, Melbourne, Australia; 5 Melbourne Children’s Sleep Centre, Monash Children’s Hospital, 246 Clayton Road, Melbourne, VIC 3168 Australia

**Keywords:** Epidemiology, Paediatrics

## Abstract

Geographic variation of paediatric tonsillectomy, with or without adenoidectomy, (A/T) has been described since the 1930s until today but no studies have investigated the factors associated with this variation. This study described the geographical distribution of paediatric A/T across the state of Victoria, Australia, and investigated area-level factors associated with this variation. We used linked administrative datasets capturing all paediatric A/T performed between 2010 and 2015 in Victoria. Surgery data were collapsed by patient residence to the level of Local Government Area. Regression models were used to investigate the association between likelihood of surgery and area-level factors. We found a 10.2-fold difference in A/T rates across the state, with areas of higher rates more in regional than metropolitan areas. Area-level factors associated with geographic variation of A/T were percentage of children aged 5–9 years (IRR 1.07, 95%CI 1.01–1.14, *P* = 0.03) and low English language proficiency (IRR 0.95, 95% CI 0.90–0.99, *P* = 0.03). In a sub-population analysis of surgeries in the public sector, these factors were low maternal educational attainment (IRR 1.09, 95% CI 1.02–1.16, *P* < 0.001) and surgical waiting time (IRR 0.99635 95% CI 0.99273–0.99997, *P* = 0.048). Identifying areas of focus for improvement and factors associated with geographic variation will assist in improving equitable provision of paediatric A/T and decrease variability within regions.

## Introduction

Tonsillectomy, with or without adenoidectomy, (adenotonsillectomy; A/T) is among the most frequently performed surgery in children, mainly indicated for obstructive sleep disordered breathing (oSDB) and recurrent tonsillitis^1,2^. Significant differences in A/T rates between regions within a country have been well documented since the 1930s when Glover was the first to observe this variation in tonsillectomy rates across English and Welsh school districts^3^. Until today, small-area geographical variation in A/T rates continues to be reported across North America^4,5^, Europe^6,7^, Asia^8^ and Africa^9^. In 2015, a government report revealed that paediatric tonsillectomy rates in Australia varied 6.5-fold across geographic areas^10^. The same report indicated that of all the states and territories, the state of Victoria had the largest variation, with a fivefold difference between the highest and lowest rates.

Small-area studies cannot resolve the question of what utilisation rate is appropriate. This would require larger national and international studies for comparison over differing paediatric populations and healthcare systems. Some variation in healthcare is warranted due to the differences in care needs between populations. However, in the defined subpopulation of children and in small-area geography, where healthcare is governed by similar policies, the magnitude of observed difference suggests much of the disparity is unwarranted, i.e. unrelated to patient need. This raises the possibility of poor access to health services, differences in the threshold for surgery between providers or patient groups and/or a lack of evidence-based guidelines. Consequently, certain children requiring A/T may not be receiving the appropriate treatment, while others are perhaps unnecessarily exposed to the risks of surgery. Understanding factors associated with the observed variation is important to guide healthcare policy and improvement initiatives in lessening the treatment gap. This study aimed to describe the geographical distribution of paediatric A/T surgery in the Australian state with the highest variation, Victoria, and to explore the area-level factors associated with this variation. We hypothesised that access to healthcare as measured by demographic factors, socio-economic status, educational attainment of the mother, English language proficiency, physical access to health services and the waiting time for surgery would be significantly associated with the rate of A/T by geographical area.

## Methods

### Ethics approval

Approval for this study and waiver for consent was granted by the Monash Health Human Research Ethics Committee. This study was performed in accordance with the guidelines and regulations of Monash Health.

### Study design and setting

We undertook a population-based cohort study using linked de-identified administrative datasets in the state of Victoria, Australia. According to the last national census in 2016, Victoria is Australia’s most densely populated state, with a total population of 5.93 million. There were 1.43 million people aged 0–19 years, of whom 1.09 million (76%) resided in the state capital, Melbourne. In Victoria, all paediatric A/T surgeries are performed in an inpatient hospital setting (none in outpatient clinics) as either a day case or with overnight post-operative stay. Across Victoria, there are 151 public hospitals and 169 private hospitals^11^. For children, there are two dedicated tertiary paediatric hospitals, both in the public sector and located in metropolitan Melbourne. Large hospitals across the state also provide secondary level paediatric care.

Geographically, Victoria is comprised of 79 Local Government Areas (LGA) which do not overlap. LGAs range in population from 2940 to 353,872 residents of all ages and in size from 861.7 to 2,208,248.3 ha^12^. In addition to the 79 LGAs, Victoria contains ten small areas with migratory populations (e.g. ski resorts and small islands) defined under “Victoria unincorporated”.

### Participants and data sources

Our study included all tonsillectomy, adenoidectomy and adenotonsillectomy surgeries performed between 1 June 2010 and 30 June 2015, at all public and private hospitals in Victoria, in patients aged between 0 and 19 years at the time of surgery. Supplied data listed age in five-year bands. Therefore, to include all paediatric patients, this study included patients aged 0–19 years inclusive.

Data were obtained from four sources: the Victorian Admitted Episodes Dataset (VAED), the Elective Surgery Information System (ESIS), the Australian Bureau of Statistics and the Public Health Information Development Unit (PHIDU).

Surgical data were drawn from the VAED and the ESIS^13,14^. Both datasets are managed by the governmental Victorian Department of Health and Human Services for administrative and billing purposes. The VAED contains information on patient demographics, hospital sector and location, and clinical details that are collected by all Victorian public and private hospitals for every hospital admission such as diagnoses and complications. The ESIS contains data on elective surgery waiting lists, including the number of days between being registered on the surgical waiting list and the surgery date. The ESIS does not contain data from private health services. Specific variables contained in the VAED and ESIS datasets were requested. The residence for each child by LGA was provided in these datasets.

Socioeconomic status for each LGA was calculated from the Socio-Economic Indexes for Areas (SEIFA), provided by the Australian Bureau of Statistics^15^. The scores of the Index of Relative Socioeconomic Disadvantage were used, which are a weighted combination of several indicators of disadvantage (e.g. income, education, employment) and standardised to a distribution with a mean of 1000 and a standard deviation of 100. An area with all of its indicators equal to the national average has a score of 1000. A higher score denotes more disadvantage, and a lower score represents less disadvantage.

Geographic barriers to accessing health care were estimated using data of residential area of remoteness, also sourced from the Australian Bureau of Statistics using the Australian Statistical Geographical Classification—Remoteness Area^16^, which defines physical access to services based on road distances from an LGA to the closest urban area with a population of 1000 or more people (urban areas of this size are assumed to have some level of healthcare service).

Health literacy data were sourced from the PHIDU^17^. The PHIDU uses data from surveys and censuses conducted by the Australian Bureau of Statistics to report sociodemographic information of geographic areas. We used two health literacy indicators summarised at the LGA level in order to be linked to the surgical data. The first was “Children in families where the mother has low educational attainment”, which described children aged less than 15 years living in families where the female parent’s highest level of schooling was year 10 or below, or where the female parent did not attend school, expressed as a proportion of all children aged less than 15 years. The second was “People aged 5 years and over who were born overseas and reported poor proficiency in English”, which described people born overseas who reported speaking English “not well” or “not at all” on the Census.

The study excluded children whose residential area was from an unincorporated area or outside of Victoria.

### Statistical analysis

Data analysis was performed in Stata (release 15.1, StataCorp). The VAED and ESIS were originally received as patient-linked datasets, with each observation describing a surgical admission. Surgery-level data were then collapsed by LGA of residence. Normality of socio-demographic factors at the LGA level was determined through the Shapiro–Wilk test and on graphical observation.

For each LGA, the observed caseload of surgery, observed case rate (per 10,000 residents) and expected caseload (age-standardised) were calculated. The observed caseload was calculated by tabulating the total number of surgeries for each LGA. The observed case rate (per 10,000 residents) was calculated by dividing the total number of surgeries for each LGA by the estimated residential population of each LGA (received from the Australian Bureau of Statistics and averaged over the five financial years of the study period 2010/11–2014/15) and multiplying by 10,000.

The caseload of each LGA was indirectly standardised for age using the state of Victoria as the standard population. To do this, cases were first stratified by age group in 5-year bands (0–4, 5–9, 10–14, 15–19 years). The caseload of each age group was divided by the estimated residential population for that age group for the whole of Victoria, producing a standardised population rate for each age group. Next, the population of each LGA was stratified by age and multiplied by the standardised population rate. These expected caseloads within each age group were then summed to produce an overall *expected caseload*.

The standardised incidence ratio (SIR) is a measure of area-level incidence likelihood and was calculated for each LGA to describe whether the number of observed cases of surgery in a particular geographic area was higher or lower than expected, given the population and age distribution for that area. The SIR was calculated as the ratio of the observed against expected cases in each LGA. The crude SIR was calculated for each LGA. While the crude SIR provides a simple measure of area-level incidence likelihood, it may be obscured by sampling variability and be imprecise for areas with small population. To reduce the impact of this variability, the confidence intervals of SIRs were calculated and areas were then grouped into: Area of significantly increased likelihood of surgery (IRR > 1, CI does not cross 1), Area of no significant difference in likelihood of surgery (CI crosses 1), and Area of significantly decreased likelihood of surgery (IRR < 1, CI does not cross 1). Choropleth maps to indicate the likelihood of surgery of LGAs were produced in ArcGIS Pro (version 2.3.0, Esri Inc.)^18^.

To identify factors associated with geographic variation, univariate and multivariable negative binomial regression models were utilised, where the outcome variable was the number of surgeries in LGAs and the independent variables were patient age, sex, residential area of remoteness, socioeconomic status, low maternal education, and low English language proficiency. These independent variables were summarised at the area-level of LGAs. Population estimates of people aged 0–19 years old in each LGA were added as the exposure variable to adjust for population size differences between areas. Outcomes of the negative binomial regression were presented as incidence rate ratios (IRR). Surgical waiting time represents a potential barrier to healthcare access^19^, but data were only available for public sector surgeries. Therefore, separate univariate and multivariable regression analyses were performed with the same independent variables, with the addition of surgical waiting time in a sub-cohort of public surgeries only. Statistical significance was defined with two-tailed tests at alpha level 0.05 (*P* < 0.05).

## Results

A total of 61,281 A/T surgeries were performed in 59,008 patients aged 0–19 years in Victoria between 2010 and 2015. After excluding patients whose residence was outside of Victoria (N = 1368) and residents of Unincorporated Victoria areas (N = 4), there was a final total sample size of 59,909 surgeries in 57,667 patients. Over the five years, there were more A/T surgeries in males (52.8%; N = 31,632) and in the younger the age group, with 37.7% (N = 22,605) of cases in 0–4 year old children, 35.8% (N = 21,455) in the 5–9 year age group, 14.2% (N = 8479) in the 10–14 year age group, and 12.3% (N = 7370) in 15–19 year age group. Half of the procedures (49.5%) were performed in private hospitals and half in public (50.5%). Table 1 provides socio-demographic summaries of the general population (not limited to children who underwent surgery) at the LGA level. In grouping areas by remoteness, there were 32 (40.5%) LGAs located within a “major city”, 33 (41.8%) LGAs in an “inner regional” area and 14 (17.7%) LGAs in an “outer regional” area. There were no Victorian LGAs classified as “remote” or “very remote”.

**Table 1:** Summary statistics of socio-demographic variables at the Local Government Area level.

	Median	IQR	Range
Population of 0–19y people (n)	10,874	3908–29,262	591–82,604
Adenotonsillectomy between 2010 and 2015 (n)	567	212–1107	14–3018
**Age group (%)**			
0–4	24.2	22.7–26.0	16.8–36.3
5–9	24.9	24.3–25.6	14.7–29.8
10–14	25.1	23.6–26.0	12.1–31.0
15–19	25.6	24.3–26.6	19.4–47.6
Male sex (%)	51.4	51.0–51.9	48.9–53.1
Socioeconomic status (score)	993	964–1026	895–1098
Low maternal educational attainment (%)	14.8	9.2–17.7	1.9–25.2
Low English language proficiency (%)	0.6	0.3–3.1	0.08–14.8

Population of 0-19y people was calculated for each Local Government Area by averaging the area’s population over five years (2010–2015).

*IQR* interquartile range.

### Incidence of surgery

**Figure 1:**
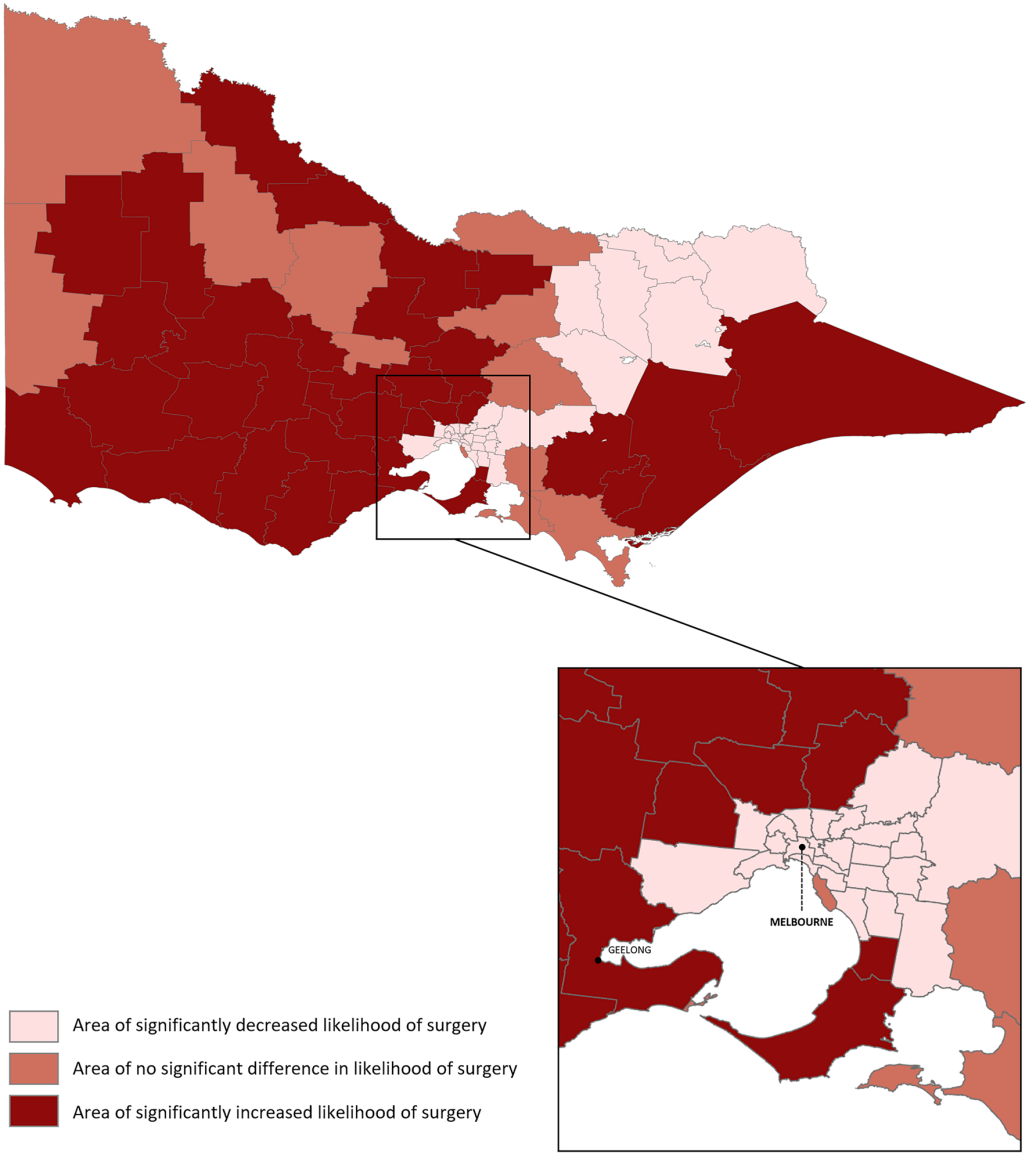
Standardised incidence ratio (SIR) of paediatric tonsillectomy, adenoidectomy and adenotonsillectomy, age standardised, by Local Government Area (LGA) in Victoria, Australia, 2010–2015. The inset map shows the city of Melbourne. Maps were generated in ArcGIS Pro (version 2.3.0, Esri Inc.)^18^.

The observed case rate of A/T over five years among LGAs ranged from 99.2 to 1014.8 per 10,000 residents aged 0–19 years, giving a 10.2-fold difference. Figure 1 shows the standardised incidence ratio of A/T surgery in Victoria. Thirty-five LGAs were identified as having a significantly increased likelihood of surgery, mostly clustered outside metropolitan Melbourne in regional areas of South-West, North, and East Victoria. Thirty-one LGAs had a significantly decreased likelihood of surgery, located in North-East Victoria, as well as central Melbourne (inset map of Fig. 1).

### Factors influencing likelihood of surgery

Area-level factors associated with increased likelihood of surgery are presented in Table 2. In univariate analyses, factors significantly associated with likelihood of surgery were a higher proportion of children aged 0–4 years, 5–9 years, 10–14 years, male sex, regional area of living (compared to metropolitan), socioeconomic status, educational attainment of the mother and English language proficiency. In the adjusted multivariable model, only the age group 5–9 and low English language proficiency were associated with likelihood of surgery. For every 1% increase in children aged 5–9 years in an LGA, there was a 7% increase in the likelihood of surgery (IRR 1.07, 95% CI 1.01–1.14, *P* = 0.03). For English proficiency, for every 1% increase in people born overseas with low English language proficiency, there was a 5% reduction in the likelihood of surgery (IRR 0.95, 95% CI 0.90–0.99, *P* = 0.03).

**Table 2:** Area-level factors influencing likelihood of adenotonsillectomy.

Area-level factor	Univariate analysis			Multivariable analysis		
	IRR	95% CI	*P*-value	IRR	95% CI	*P*-value
**Age group**						
0–4	0.97	0.94–1.00	0.03^*	0.99	0.95–1.03	0.67
5–9	1.08	1.02–1.15	0.01^*	1.07	1.01–1.14	0.03*
10–14	1.06	1.02–1.10	0.004^*	0.98	0.92–1.05	0.61
15–19	0.98	0.95–1.02	0.31	Omit	–	–
Male sex	1.12	1.00–1.25	0.047^*	1.07	0.96–1.19	0.23
**Area of remoteness**						
Major city	Reference	–	–	Reference	–	–
Inner regional	1.52	1.26–1.83	< 0.001^*	1.05	0.77–1.45	0.74
Outer regional	1.43	1.12–1.82	0.004^*	1.03	0.69–1.53	0.89
Socioeconomic status	1.00	0.99–1.00	< 0.001^*	1.00	0.99–1.00	0.43
Low maternal education	1.03	1.01–1.05	< 0.001^*	1.01	0.97–1.04	0.66
Low English language proficiency	0.93	0.91–0.96	< 0.001^*	0.95	0.90–0.99	0.03*

^Denotes *p*-value < 0.1 for univariate analysis for further inclusion into the multivariable model.

*Denotes significance at *p*-value < 0.05.

*IRR* incidence rate ratio.

Surgical waiting time was investigated in a separate model, using data for patients of public hospitals only (Supplementary Table S1 online). In the univariate model, surgical waiting time was not a significant predictor of surgery rate (IRR 0.998 95% CI 0.995–1.001, *P* = 0.243). However, when adjusted for the same factors in Table 1, surgical waiting time was significantly associated with likelihood of surgery, with every one day increase in the median wait time in a LGA there was a 1% reduction in the likelihood of surgery (IRR 0.99635 95% CI 0.99273–0.99997, *P* = 0.048). In this subpopulation analysis, maternal low educational attainment was also significantly associated with an increase in likelihood of surgery in both univariate (IRR 1.06, 95% CI 1.03–1.10, *P* < 0.001) and multivariable analysis (IRR 1.09, 95% CI 1.02–1.16, *P* < 0.001). However, percent of children aged 5–9 years and English language proficiency were not significant factors in this subpopulation analysis.

## Discussion

Our study describes substantial geographic variation in paediatric A/T surgery in the state of Victoria. To our knowledge, this study is the first to explore the associations of geographic variation in A/T surgery. We found area-level percent of age group 5–9 years, English language proficiency, maternal educational attainment, and surgical waiting time to each have a small impact on the likelihood of surgery.

In identifying associations of the observed geographic variation, we found that higher percent of children aged 5–9 years in an area increased the likelihood of surgery. This is unsurprising, given that recurrent tonsillitis and oSDB are common in school-aged children due to close-contact infection in schools and growth of lymphoid tissue around the airway during these years. We did not find general socioeconomic status to be directly associated with likelihood of surgery, but found that higher rates of low English language proficiency decreased surgical likelihood and, in the subpopulation of public patients, higher rates of low maternal educational attainment increased likelihood of surgery. Although we did not measure ethnicity directly, based on previous work suggesting higher rates of oSDB in non-Caucasian populations^20^, we would have expected higher rates of surgery in areas with lower English language proficiency but found the reverse. While language barriers have not been studied in relation to A/T surgery, it is well described that in English speaking countries, children in non-English primary language households experience multiple disparities in general health, access to care and use of health services^21^. A combination of factors may explain this, including difficulty in seeking and lack of knowledge of healthcare options, and cultural differences in perception of healthcare-seeking in caregivers^22^. Given this finding, future efforts in reducing variation should target better information on access to healthcare in languages other than English and increase community awareness of the importance of seeking appropriate medical care for children with tonsillitis and oSDB. Studies have additionally linked low health literacy (estimated by maternal educational attainment in this study) with increased use of general healthcare services and emergency department use^23,24^. Caregivers with low health literacy are less likely to be appropriately informed in the decision-making process. Consequently, such caregivers may not understand the argument if watchful waiting, instead of surgery, is recommended based on current guidelines^1^. Guidelines recommend surgery for severe oSDB or for frequent recurrent tonsillitis meeting the Paradise criteria (seven or more episodes of acute tonsillitis in the preceding year, or five or more in each year for two years, or three per year for three years). Research studying other types of surgery have found that implementation of decision aids significantly reduced overall surgery rates and variation as they increased consistency between patients’ knowledge of the benefits and risks of the treatment options^25^. Decision aids are tools that explicitly present the factors behind the healthcare decision that needs to be made, provide information about the options and outcomes, and support informed discussion between patients and healthcare providers. Future studies should therefore develop and assess the effect of treatment decision aids on paediatric A/T surgery rates. Patients’ low educational attainment can additionally influence physicians’ perceptions and clinical decision making. Research has shown that physicians are less likely to perceive low SES patients as responsible, intelligent, able to comply with medical advice and return for follow up visits^25^. In Australia, which has a gatekeeping system where GPs decide on referral to specialists, many factors may be influencing the decision to refer to diagnostic testing and surgical treatment for oSDB and tonsillitis. Our finding of higher likelihood of surgery in areas with higher rates of low maternal educational attainment could be explained by physicians wanting to limit repeated follow-up visits for conservative treatment and refer to surgery for convenience.

Additionally, we found that a longer median surgical waiting time in an area reduced the likelihood of surgery. This factor has not been previously studied, but importantly highlights a “supply-sensitive” aspect that contributes to surgical variation. Supply-sensitive care is a concept termed by Wennberg^26^, which describes uneven resource distribution as a cause of variation.

We found a tenfold difference between the lowest and highest rates of surgery in Victoria. Some level of disparity is expected, but this large level of variation likely indicates inequity and/or inefficiency in the provision, access and use of surgical services. As previously mentioned, this variation in Victoria was shown in the *Australian Atlas of Healthcare Variation* in 2015^10^. Our study utilised data over five years instead of one, and used a smaller geographical unit, allowing for more focused results. This use of the smaller Local Government Areas may explain our finding of a tenfold difference in geographical rate, compared to the fivefold difference reported for Victoria by the Atlas using the larger “statistical area 3” geographical unit. Our study confirms the previous finding of high surgery rates in regional Victoria compared to metropolitan Melbourne. An exception to this is the lower rates of surgery in North-East Victoria. However, the lower rates can be explained by the closest hospital for this region being located in the neighbouring state of New South Wales, which our data does not capture. Higher rates of A/T in less urbanised areas has been previously documented across Australia^10^, as well as in the United States and Canada^4,27^. One explanation could be that many patients living in regional areas need to travel long distances to hospitals, particularly those with specialised paediatric services. This difficulty in accessing healthcare may paradoxically cause an increase in the number of A/T surgeries aimed at limiting repeated visits to a general practitioner for conservative treatment. Therefore, prompt surgery may be recommended for convenience. Another reason could be higher incidences of tonsillitis and oSDB in rural and regional areas, but this seems unlikely given the relative homogeneity of the Victorian population and geographical climate. Limited studies of variation in disease prevalence exist and none have been conducted specifically in Victoria^28^.

Overall, the factors we found that were significantly associated with the rate of surgery in a LGA only contributed a small amount to the observed variation due to the relatively low IRR values. Additional likely explanations of geographic variation in general relate to the clinical decision-making process. Glover, who first reported geographical variation in tonsil and adenoid surgery 80 years ago, rejected an aetiological association and purported that “these great variations on local incidence…appear to depend almost entirely upon medical opinion in the individual area”^3^. This type of “preference-sensitive care”, as termed by Wennberg^26^, describes healthcare provision being dependent on the opinions of physicians and patients. In Glover’s study, tonsillectomy rates in specific school districts changed rapidly as one health officer (responsible for diagnosis and referral of school children for surgery) was replaced with another. Forty years later, a study by Bloor et al. also suggested surgeons as well as referring physicians influenced varying tonsillectomy rates in Scotland^29,30^. They observed and interviewed surgeons from areas with low and high rates of tonsillectomy for recurrent tonsillitis, and found differences in clinical-care decisions. Surgeons in areas with higher rates of surgery placed importance on physical examination in the decision-making process, whereas surgeons from districts with lower rates tended to rely more on the patient’s medical history. In the contemporary context, variation in rate across areas may be influenced by the referral practices of general practitioners and surgeon attitudes regarding the disease threshold for surgery. For recurrent tonsillitis, recent guidelines advise surgery based on tonsillitis frequency and severity as per the Paradise criteria^31^. However, for oSDB there is currently no clearly defined threshold for surgery. There is also limited research investigating the cost-benefits of surgery for oSDB, although evidence from Israel would suggest reduced healthcare utilisation after A/T in children with oSDB^32^. Moreover, guidelines for A/T for oSDB depend on defining the severity of oSDB. The gold-standard for determining the severity of oSDB is polysomnography, which is costly, has a long waiting time and is not available at all health centres. In addition, metrics of severity of oSDB on polysomnography are poorly correlated with measures of morbidity and also poor predictors of benefit of surgery^33^. Therefore, the lack of a practical simple diagnostic tool for oSDB and a clearly defined threshold at which surgery is indicated results in a large grey area of clinical discretion.

Another explanation for geographical discrepancy may be “supply-sensitive care” factors such as disproportionate availability of paediatric hospitals and/or otolaryngologists in certain areas. Currently no research has investigated this association for A/T surgery. However, research has found that the density of physician workforce is not a large contributor to geographic variation^34^. It found that high rates of procedures were not due to the number of surgeons in a specific area, but rather, similar to Glover’s findings, by enthusiasm for that procedure among a small number of high-volume surgeons in that region. This is consistent with our findings given that metropolitan Melbourne has the greatest density of hospitals and healthcare providers but amongst the lowest rates of surgery.

A key strength of our study was that it comprised a complete capture of all paediatric A/T surgery in Victoria over a 5-year period. Compared to the description in the *Australian Atlas of Healthcare Variation*^10^, which used data over a one-year period, our larger dataset likely minimised random errors and population sampling bias, especially for areas with small paediatric populations. It also included adenoidectomy data as well as tonsillectomy, and highlights a very similar pattern of variation. A limitation of this study was the lack of available surgical waiting time data for surgeries performed at private hospitals, meaning that this sub-analysis had to be confined to public hospital patients, which accounted for half the entire cohort. Furthermore, to strengthen the investigation of factors associated with geographic variation, additional approaches would be required such as a mixed-methods study with qualitative aspects to understand patient/provider preference in decision making and other factors influencing access to healthcare at the individual rather than population level. An individual-level analysis was also not possible due to the lack of data of children who had not undergone A/T in Victoria for comparison.

## Conclusions

We have described regional variation in paediatric A/T surgery across a populous Australian state, finding that surgery is more likely to be performed in those living in regional than metropolitan areas. We also identified geographic variation of A/T surgery to be associated with percent of children aged 5–9 years, English language proficiency, maternal educational attainment and surgical waiting time in a given LGA. These findings provide areas of focus for improvement in the provision of A/T surgery, including further research and quality improvement work that will help improve access to paediatric A/T surgery to ensure consistency and decrease variability across geographic areas.

## Supplementary Information

Supplementary Information.

## Data Availability

The data that support the findings of this study are available from the Victorian Agency for Health Information but restrictions apply to the availability of these data, which were used under license for the current study, and so are not publicly available. Data are however available upon request from the Victorian Agency for Health Information.
