# Supplementary material for: Sociodemographic associations of geographic variation in paediatric tonsillectomy and adenoidectomy
Source: Sci Rep. 2021 Aug 5;11:15896. doi: 10.1038/s41598-021-95522-5 (PMC8342528; doi:10.1038/s41598-021-95522-5)
Supplement: Supplementary file 1 — Supplementary Information. [file 41598_2021_95522_MOESM1_ESM.pdf]

SOCIODEMOGRAPHIC ASSOCIATIONS OF GEOGRAPHIC VARIATION IN PAEDIATRIC TONSILLECTOMY AND ADENOIDECTOMY

Aimy HL Tran, Danny Liew, Rosemary SC Horne, Joanne Rimmer, Gillian M Nixon

SUPPLEMENTARY MATERIAL

Table S1. Sub-analysis of area-level factors influencing likelihood of adenotonsillectomy

	Univariate Analysis			Multivariable Analysis		
**Area-level Factor**	**IRR**	**95% CI**	**P-value**	**IRR**	**95% CI**	**P-value**
Median surgical waiting time (days)	0.998	0.995 - 1.001	0.243	0.99635	0.99273 - 0.99997	0.048
Age group						
0-4	1.03	0.97 - 1.09	0.36	1.05	0.96 - 1.14	0.27
5-9	1.06	0.95 - 1.18	0.27	0.97	0.87 - 1.08	0.59
10-14	0.98	0.90 - 1.06	0.57	1.03	0.92 - 1.16	0.60
15-19	0.97	0.92 - 1.02	0.23	Omitted	–	–
Male sex	1.002	0.80 - 1.26	0.987	0.93	0.75 - 1.16	0.52
Area of remoteness						
Major city	Reference	–	–	Reference	–	–
Inner regional	1.31	0.93 - 1.86	0.13	0.80	0.46 - 1.39	0.43
Outer regional	0.61	0.39 - 0.98	0.04	0.31	0.15 - 0.65	0.00
Socioeconomic status	0.995	0.991 - 0.999	0.01	0.998	0.990 - 1.007	0.66
Low maternal education	1.06	1.03 - 1.10	<0.001	1.06	1.00 - 1.12	0.07
Low English language proficiency	0.99	0.93 - 1.04	0.66	0.95	0.87 - 1.04	0.26

This is a sub-analysis of the full cohort, only including public patients
Abbreviations: IRR=incidence rate ratio
Age group 15-19 was omitted due to collinearity.
